# Psychology in Virtual Reality: Toward a Validated Measure of Social Presence

**DOI:** 10.3389/fpsyg.2021.705448

**Published:** 2021-10-04

**Authors:** Radosław Sterna, Katja Zibrek

**Affiliations:** ^1^Emotion and Perception Lab, Faculty of Philosophy, Institute of Psychology, Jagiellonian University, Krakow, Poland; ^2^MimeTIC Team, Inria, Université de Rennes, CNRS, IRISA, Rennes, France

**Keywords:** VR, virtual reality, social presence, agent, copresence

## 1. Introduction

Since its introduction as a methodological tool to study social behavior (Blascovich et al., 2002), Virtual Reality (VR) has been seen as a promising instrument which might facilitate dealing with some of the psychological experiments' pitfalls by increasing reproducibility, ecological validity and experimental control (Pan and Hamilton, 2018). These advantages prompted more and more psychologists to implement VR into their research on social behavior where the responses to virtual characters are studied.

One of the major factors leading to an effective social VR simulation is social presence, which can simply be described as the “sense of being with another” (Biocca et al., 2001). It describes the ability of the VR system to create the illusion that the user is inhabiting the virtual environment with someone else. The construct can be perceived as a prerequisite of the ecologically valid simulation of social interaction: if the user feels social presence with another in VR, he or she will exhibit behavior similar to a real interaction. In this context, social presence can be seen as a baseline necessary for social influence induction (Discussion: Swinth and Blascovich, 2002).

However, the research on social presence suffers from several flaws. Firstly, there is a terminological disagreement among the researchers which transfers into different operationalizations of the construct (Oh et al., 2018). Secondly, the terminological inconsistency causes measurement difficulty. How to measure the construct? What are we actually measuring? Thirdly, some researchers (e.g., Bailenson et al., 2001, 2003) used behavioral or psychophysiological responses as markers of social presence. However, these measures have not been properly validated yet either.

In the current opinion paper, we aim to discuss the issues related to the social presence construct and its measurement, and why it is important to remain cautions when conducting research or interpreting results related to social presence. We also give suggestions on how to approach the described problems, by giving concrete examples from good practice.

## 2. The Problem of Defining Social Presence

Virtual reality is a highly immersive medium that can create a believable illusion of being physically present in an artificial environment with another, who is typically presented in a virtual character form. The terms social presence or co-presence commonly describe this experience, and are sometimes used interchangeably (Bailenson et al., 2005) while other times they describe distinct constructs (Nowak, 2001) or subdimensions (Harms and Biocca, 2004). In addition, concepts such as plausibility illusion (Slater, 2009), and positive affect (“warmth,” in Algharabat et al., 2018) are used, which are related to the construct of social presence but also have distinct qualities and applications. For example, plausibility illusion is a general sense that a scenario in VR is believable, but this scenario does not need to include a virtual character. A higher social presence with the virtual character could also induce a positive reaction from the user, e.g., the character appears warm and welcoming. However, this interpretation may not include all types of virtual interactions in complex scenarios.

Furthermore, there are divisions in terms of definition, particularly whether social presence is mostly a cognitive or behavioral phenomena. The first one warrants the use of questionnaires, asking directly about the sense of being with another (Harms and Biocca, 2004), while the other relies on indirect measures, such as eye-tracking and psychophysiology (Slater et al., 2009), social influence (Slater et al., 2006), or choice making (Skarbez et al., 2017; Murcia-López et al., 2020). It is therefore not surprising that the studies in social VR used different terminology of social presence, which limits the comparability of their results.

A proposed meta-review of social presence by Cummings and Wertz (2018), is planning to address the problem of operationalization of social presence by including the differing conceptualizations as dependent variables in the analysis of the predictors of social presence. This would indicate whether some features more directly relate to particular social presence conceptualizations.

Another example which could help solving the terminological confusion in the field of social presence is described in the initiative by Fitrianie et al. (2020). In the mentioned initiative, independent researchers in the field of artificial virtual agents, were asked to sort semantically overlapping concepts into a reduced set of groups. These concepts were taken from several studies, which were used when describing artificial agents. This helped identifying unifying set of constructs, and create the basis for a future questionnaire. We believe that such an initiative would be beneficial in the field of social presence as well.

Before experimental investigation will provide conclusions and the consensus will be reached we advise researchers to make use of definitions included in the meta-analytical studies (e.g., Oh et al., 2018). This practice increases comparability of the individual study outcomes with the existing literature.

## 3. The Problem of Direct Measures of Social Presence

In the review on social presence Oh et al. (2018) list the questionnaires used in the studies on this construct (Table 2, p. 11–14). Roughly counting, there are over 40 different questionnaires mentioned. Intelligent Virtual Agents (IVA) researchers recently noted that there is a continuous practice in the community of creating new questionnaires instead of reusing existing ones (Fitrianie et al., 2020, p. 1). Moreover, only a minority of the social presence questionnaires which are in use were properly psychometrically validated (rare exceptions, e.g., Harms and Biocca, 2004; Poeschl and Doering, 2015) and usually the tools are constructed *ad-hoc* for particular research purposes (Table 2, Oh et al., 2018). Not only do these questionnaires create unreliable measures, they also prevent comparison between different studies.

Reasons why researchers do not use validated questionnaires are probably due to the fact they do not fit the various experimental contexts in which the measuring of social presence occurs (Biocca et al., 2003). Therefore, the eventual tool to be chosen would need to be general-purpose i.e., it would not consist of task specific questions which can prove useful only in certain experimental designs (e.g., public speaking scenario: “The people's behavior influenced my style of presentation.” Poeschl and Doering, 2015). One of the examples of the well-constructed questionnaire is Networked Minds Measure of Social Presence (Biocca et al., 2001) which consists of task-neutral questions [e.g., “(My partner's) presence was obvious to me.”] and was psychometrically tested (Harms and Biocca, 2004). We encourage the community to strive for the unification not only in concepts, but also in measures used. In other words, what we propose is the strive for a standard (i.e., validated, accepted and used by the community; Skarbes et al., 2021) measure of social presence by revisiting already existing/developing new questionnaires, validating them and promoting their usage. Before it happens, individual researchers wanting to test social presence in their experiments would be encouraged to make use of psychometrically tested tools such as the aforementioned.

## 4. The Problem of Indirect Measures of Social Presence

VR allows for measurement of a multi-level indirect response: ranging from unconscious physiological changes, through semi-conscious responses, such as proximity behavior, to conscious volitional actions (Slater et al., 2009). A popular behavioral measurement of social presence is the proximity (Bailenson et al., 2001, 2003, 2005). The notion behind it is that people will keep similar distances to virtual characters in VR as they do with real people when social presence is sufficiently high. For example, similar distance patterns which exist in real life were discovered in VR when assessing, e.g., the effect of age and gender (Iachini et al., 2016), attractiveness (Zibrek et al., 2020), and facial expression (Bönsch et al., 2020). Other indirect measures include eye-tracking and psychophysiological measurements (Slater et al., 2009).

The indirect measures also have certain disadvantages. One of the issues related to the indirect measurements is that the meaning of behavior or physiological signal is open to debate (Cummings and Bailenson, 2016).

The studies investigating social presence lack psychometrical validation of the indirect measures, which is especially problematic with behavioral measures and their reliability (Dang et al., 2020). In addition, some behavioral measures of social presence, such as proximity (the distance between the user and the virtual agent) are used but not properly controlled for the confounding factors, such as characters' emotional expression (Zibrek et al., 2019), gender of the participant and the virtual agent (Iachini et al., 2016), etc. With proper control of experimental conditions, however, the problem of confounding factors can be minimized. For example, a proximity experiment can include a control condition in the physical world with real obstacles (Sanz et al., 2015).

If we want to properly use the indirect measures of social presence, they need to be tested for their psychometric properties (example: Meehan et al., 2005), otherwise, we might fall into a trap of falsely assuming an isomorphic relationship between the physiological signal or behavior and social presence (Cacioppo and Tassinary, 1990) as well as reduce the comparability of the studies.

As discussed in 2, the terminology problem represents an obstacle to the validation of measures and this holds true for indirect measures also. The researchers stressing the importance of behavioral aspects of social presence (e.g., Bailenson et al., 2004) might put more emphasis on the comparisons between real-life behavior (e.g., proximity) and the behavior in the simulation as it was done with proximity measurement which originated from real-life observations of Hall (1966). They may also consider designing validations similar to those used in presence studies where breaks-in-presence (BIP; Slater et al., 2003) are correlated with the signal (psychophysiology) or behavioral responses. On the other hand, the researchers who perceive social presence as a cognitive phenomenon (e.g., Harms and Biocca, 2004), would put more emphasis on correlating the indirect measurement with questionnaires. In our view, these two perspectives are not exclusive and both can be used as mediums to validate indirect measures (see example: Meehan et al., 2005).

Moreover, recent research Van Kerrebroeck et al., 2021) shows the unique possibility of VR to not only track a position of the body in space, but also to quantify bodily and multisensory (e.g., eye contact, speech) patterns of interaction and combine them with performance analysis as well as subjective measurement, creating a compound index of social presence.

## 5. Discussion

Our work is part of a larger trend making VR technology more accessible to the social scientists (e.g., Fox et al., 2009; Parsons et al., 2017; Pan and Hamilton, 2018; Gonzalez-Franco et al., 2020) and aimed to present our viewpoint on some of the issues related to social presence understanding and measurement.

In our paper, we give some concrete solutions and future directions for researchers which would benefit the field of social presence:

- the research initiative, similar to Fitrianie et al. (2020) to approach the problem of terminology;
- encourage researchers to use existing validated questionnaires of social presence, whenever possible;
- to consider adding indirect measures to their experiments and have a proper control of the confounding factors.

Related to the first suggestion, we realize that this requires a community effort which can be timely and constantly changing as new findings emerge. Nevertheless, it would help address the confusion which exists in terminology and provide a good basis for the interpretation of the existing research and help with the construction of a standardized instrument to evaluate social presence from the findings which were already gathered but not integrated. In order to reduce the effort, the researchers should therefore, when they can, use existing questionnaires of social presence. Finally, while indirect measures do have their shortcomings, we recommend they be added as part of the measures in an experiment design in the investigation of social presence.

We hope that researchers will assume a cautious approach when interpreting results of social presence in their own experiments, as the field is still developing. On the other hand, social presence as a relatively new and ongoing field of research, provides an exciting venue for any researcher interested in exploring interactive virtual environments.

## Author Contributions

RS and KZ wrote the manuscript, contributed to the conception of the manuscript, and performed the article search. Both authors listed have made substantial intellectual contribution to the work, revised the manuscript, read, and approved the submitted version.

## Funding

This work was financed from the budget for science in the years 2019–2021 as a research project The impact of virtually generated characters: human aspects of bots (project number: DI2018 015848) under the Diamond Grant programme financed by the Ministry of Education and Science of Poland. The research has been supported by a grant from the Priority Research Area (FutureSoc) under the Strategic Programme Excellence Initiative at the Jagiellonian University.

## Conflict of Interest

The authors declare that the research was conducted in the absence of any commercial or financial relationships that could be construed as a potential conflict of interest.

## Publisher's Note

All claims expressed in this article are solely those of the authors and do not necessarily represent those of their affiliated organizations, or those of the publisher, the editors and the reviewers. Any product that may be evaluated in this article, or claim that may be made by its manufacturer, is not guaranteed or endorsed by the publisher.
